# Alnus hirsuta (Spach) Rupr. Attenuates Airway Inflammation and Mucus Overproduction in a Murine Model of Ovalbumin-Challenged Asthma

**DOI:** 10.3389/fphar.2021.614442

**Published:** 2021-02-10

**Authors:** Ba-Wool Lee, Ji-Hye Ha, Yeongseon Ji, Seong-Hun Jeong, Ju-Hong Kim, Jihye Lee, Ji-Young Park, Hyung-Jun Kwon, Kyungsook Jung, Jong-Choon Kim, Young-Bae Ryu, In-Chul Lee

**Affiliations:** ^1^ Functional Biomaterial Research Center, Korea Research Institute of Bioscience and Biotechnology, Jeongeup-si, South Korea; ^2^ Department of Veterinary Pharmacology and Toxicology, College of Veterinary Medicine, Chonnam National University, Gwangju, South Korea

**Keywords:** allergic asthma, airway inflammation, mucus overproduction, mitogen-activated protein (MAP) kinase, nuclear factor-kappa B, *Alnus hirsuta* (spach) Rupr.

## Abstract

*Alnus hirsuta* (Spach) Rupr. (AH), a member of the Betulaceae family, is widely used in Eastern Asia of as a source of medicinal compounds for the treatment of hemorrhage, diarrhea, and alcoholism. In this study, we investigated the protective effects of a methanolic extract of AH branches against airway inflammation and mucus production in tumor necrosis factor (TNF)-α-stimulated NCI-H292 cells and in an ovalbumin (OVA)-challenged allergic asthma mouse model. Female BALB/c mice were injected with OVA (40 μg) and aluminum hydroxide (2 mg) on days 0 and 14 to induce allergic airway inflammation. The mice were then challenged with 1% OVA from days 21–23. Mice were treated with AH (50 and 100 mg/kg/day; 2% DMSO) or dexamethasone (positive control; 3 mg/kg/day) from days 18–23. AH treatment effectively attenuated airway resistance/hyperresponsiveness and reduced levels of T helper type 2 (Th2) cytokines, eotaxins, and number of inflammatory cells in bronchoalveolar lavage fluid, and immunoglobulin E in serums of OVA-challenged mice. In histological analysis, AH treatment significantly inhibited airway inflammation and mucus production in OVA-challenged mice. AH treatment downregulated the phosphorylation of I kappa B-alpha, p65 nuclear factor-kappa B (p65NF-κB), and mitogen-activated protein kinases with suppression of mucin 5AC (MUC5AC) in lung tissue. Moreover, AH treatment decreased the levels of pro-inflammatory cytokines and Th2 cytokines, as well as MUC5AC expression, and inhibited the phosphorylation of p65NF-κB in TNF-α-stimulated NCI-H292 cells. These results indicate that AH might represent a useful therapeutic agent for the treatment of allergic asthma.

## Introduction

Allergic asthma is a widespread global health issue characterized by chronic inflammatory disease mediated through eosinophilic inflammation, mucus overproduction, elevated allergen-specific immunoglobulin E (IgE), and airway hyperresponsiveness (LaVan et al., 2002; Park et al., 2016). It is caused by inhalation of house dust, pollen, animal dander and smoking, leading to an immune imbalance via elevation of T helper type 2 (Th2) cells in the airway (Calderon et al., 2013; Perret et al., 2013). Th2 cytokines, such as interleukin (IL)-4, IL-5, and IL-13, are related to the development and maintenance of allergic airway inflammation (Peebles and Aronica, 2019). Th2 cytokines recruit eosinophils and induce their infiltration and activation, as well as stimulate B cells that produce allergen-specific IgE. In particular, activated eosinophils contribute to persistent inflammation, airway hyperresponsiveness, and mucus overproduction in allergic asthma (Schatz and Rosenwasser, 2014; Lee et al., 2017). Currently, many researchers have demonstrated that the suppression of Th2 cytokines is an important target for controlling allergic asthma (Leon, 2017; Gandhi et al., 2019).

Nuclear factor-kappa B (NF-κB) is a transcription factor that plays a central role in modulating inflammatory responses (Zhang et al., 2017). Various stimuli activate NF-κB, after which it translocate into the nucleus and binds DNA to regulate the transcription of genes involved in asthmatic responses, including those for Th2 cytokines and mucin 5AC (MUC5AC) (Cai et al., 2018; Tian et al., 2019). The mitogen-activated protein kinases (MAPKs), such as c-Jun N-terminal kinase (JNK), extracellular signal-regulated kinase (Erk), and p38MAPK, are regarded as critical regulators of inflammation (Kim et al., 2019). In allergic asthma, the phosphorylation of MAPKs activates various transcription factors, leading to the production of proinflammatory cytokines, chemokines, and mucins (Alam and Gorska, 2011; Chauchan et al., 2018). Therefore, suppression of NF-κB and MAPK phosphorylation may constitute effective therapeutic targets for the treatment of allergic asthma. Currently, asthmatic patients are commonly treated with β_2_-agonists, bronchodilators, and corticosteroids. However, these treatments have not been successful in all patients and can cause numerous adverse effects such as growth inhibition and gastric ulcer formation (Ciriaco et al., 2013; Wise, 2014). Thus, in recent years, many researchers have investigated natural products as alternative medicines that are safer and more effective in treatment of asthma (Bang et al., 2015; Lee et al., 2019).

*Alnus hirsuta* (Spach) Rupr. (AH) is a deciduous tree that belongs to the Betulaceae family. It is widespread in Eastern Asia. It is used in traditional medicine for treatment of fever, diarrhea, hemorrhage, and alcoholism (Lee, 1996). Previous studies reported the beneficial effects of AH extract, including its antioxidant and anti-inflammatory activities through decreased production of reactive oxygen species (ROS) and nitric oxide (NO), and increased DPPH radical scavenging activity in lipopolysaccharide (LPS)-stimulated RAW264.7 cells (Hu and Wang, 2011). Moreover, an AH extract also inhibited cyclooxygenase (COX)-2, inducible nitric oxide synthase (iNOS) and tumor necrosis factor (TNF)-α production, through downregulation of NF-κB phosphorylation in LPS-stimulated RAW264.7 cells (Jin et al., 2007). However, the effects of an extract of AH branches on airway inflammation in allergic asthma have not been investigated. Thus, we evaluated the inhibitory potential of AH on airway inflammation in TNF-α-stimulated NCI-H292 cells and an ovalbumin (OVA)-challenged asthmatic mouse model. To investigate the therapeutic mechanism of AH, we measured the expression levels of Th2 cytokines and mucus production via MAPKs/p65NF-κB in the OVA-challenged asthmatic mouse model.

## Materials and Methods

### UPLC Q-TOF/MS Analysis

The methanol extract from the branch of *A. hirsuta* (Spach) Rupr. (AH), which belongs to the Betulaceae family, was collected from Gangwon Province (Gangwon-do, South Korea) and obtained from The Korea Plant Extract Bank of Korea Research Institute of Bioscience and Biotechnology (KRIBB, PB2357.6). The tentative identification of compounds in AH was analyzed by ACQUITY UPLC system coupled with Vion IMS QToF mass spectrometer (Waters Corp., Milford, MA, United States) using BEH C18 column (2.1 × 100 mm, 1.7 μm) and two mobile phases, 0.1% formic acid in water (A) and acetonitrile (B). The column and sample tray temperature were maintained at 35°C. The flow rate was 0.4 ml/min and elution conditions were optimized as follows: 0–1 min, 5% B; 1–20 min, 5–100% B; 20–20.30 min, 100% B; 20.30–22.40 min, 100–5% B; 22.40–25 min, 5% B. The mass spectrometer operated in negative mode from 100 to 1,500 Da with a 0.2 s scan time using a desolvation temperature of 350°C with desolvation gas flow of 800 L/h (N_2_), source temperature of 110°C, and cone voltage of 40 V. Leucine-enkephalin was used as the lock mass ([M–H]– *m/z* 554.2615). The full scan data and MS/MS spectra were acquired using a UNIFI software (Waters Corporation, Milford, MA).

### Test Compound and Reagents

The human lung epithelial cell line, NCI-H292, was purchased from American type culture collection (ATCC; Rockyville, MD, United States). Ovalbumin (OVA) and dexamethasone (DEX) were purchased from Sigma-Aldrich (St. Louis, MO, United States) and human recombinant tumor necrosis factor alpha (TNF-α) was obtained from Peprotech (Rocky Hill, NJ, United States). The enzyme-linked immunosorbent assay (ELISA) kits for IL-4, IL-5, IL-6, IL-13, TNF-α and eotaxin (R&D system, Minneapolis, MN, United States), MUC5AC (Cusabio Biotech Co., Wuhan, China) and IgE (BioLegend, CA, United States) were used according to the manufacturer’s instructions. The Diff-Quik^®^ stain kit and Periodic acid-Schiff (PAS) kit were purchased from IMEB (San Marcos, CA, United States).

### Experimental Procedure

Female BALB/c mice (specific pathogen-free, 7 weeks old, 20–25 g, Orient Bio, Seongnam, South Korea) were housed in groups of three or four under standard conditions (temperature 22 ± 2°C, humidity 55 ± 5%, 12 h light/dark cycle), and provided sterilized water and standard rodent chow (Orient Bio). The Institutional Animal Care and Use Committee of the Korea Research Institute of Bioscience and Biotechnology approved the protocols for the animal study (Approval number: KRIBB-AEC-18234).

Specific pathogen-free female BALB/c mice were randomly divided into the following five groups (n = 7 per group).

- Normal control (NC) group: treated with vehicle (2% DMSO) from days 18 to day 23 and phosphate-buffered saline (PBS) sensitization/challenge.
- OVA group: treated with vehicle (2% DMSO) from day 18 to days 23 and OVA sensitization/challenge.
- DEX group: treated with dexamethasone (3 mg/kg) from day 18 to days 23 and OVA sensitization/challenge.
- AH50 group: treated with AH (AH 50 mg/kg; 2% DMSO) from days 18 to day 23 and OVA sensitization/challenge.
- AH100 group: treated with AH (AH 100 mg/kg; 2% DMSO) from days 18 to day 23 and OVA sensitization/challenge.

OVA-challenged allergic asthma model was performed while using the method of Shin et al. (2019a). Mice were sensitized with 40 μg OVA with 2 mg of aluminum hydroxide gel by intraperitoneal injection on days 0 and 14. At days 18–23 after initial sensitization, mice were administered the treatments once daily by oral gavage. At the time of oral administration, mice were challenged for 1 h with OVA (1%, w/v, in PBS) using an ultrasonic nebulizer (NE-U12; OMRON Corp., Tokyo, Japan) from days 21–23. Dexamethasone was used as the positive control and was administered to mice at a dose of 3 mg/kg body weight (Shin et al., 2019a). The potential effects of AH on animal models have not been elucidated. Then, we referred to anti-inflammatory activity of *Alnus* genus in endotoxic shock model (Saxena et al., 2016). In addition, several studies used to 50 or 100 mg/kg of doses to investigate herbal medicine for controlling of allergic asthma (Costa et al., 2012; Shin et al., 2015; Shin et al., 2017). Thus, we selected the doses of AH 50 and 100 mg/kg.

### Measurement of Airway Resistance and Airway Hyperresponsiveness

Airway resistance and airway hyperresponsiveness were evaluated by Flexivent (SCIREQ Scientific Respiratory Equipment Inc., Montreal, PQ, Canada) (Shalaby et al., 2010) 24 h after the last OVA challenge. After baseline measurements of impedance (Zrs), each animal was exposed to methacholine (5, 10 and 20 mg/ml) or PBS aerosols via a nebulizer (Aeroneb; SCIREQ) for 10 s levels of airway resistance and airway hyperresponsiveness were measured every 30 s for 1 min and refreshed for 2 min. The level of airway hyperresponsiveness was calculated by Flexivent system. The volume history of the lung was established with 6 s deep inflations to a pressure limit of 30 cmH_2_O.

### Analysis of Bronchoalveolar Lavage Fluid Collection

BALF collection was performed with the method of Shin et al., (2015). Briefly, to obtain BALF, ice-cold PBS (0.7 ml) was infused into the lungs two times and withdrawn each time with a tracheal cannula (total volume, 1.4 ml). The collected BALF was centrifuged at 1,000 rpm for 10 min at 4°C. The supernatants were collected and stored at −70°C before cytokine analysis. The cell pellet was re-suspended with 500 μL ice-cold PBS and attached on a slide using a Cytospin 4 centrifuge (Thermo Scientific, Waltham, MA, USA) (1,000 rpm, 5 min, 20°C). Differential cell count was performed with Diff-Quik® staining reagent according to the manufacturer’s instructions. Five images of each slide were captured with a Leica DM5000B microscope and the Leica Application Suite acquisition software (Leica Microsystems, Wetzlar, Germany) under 40× objective lens. Thereafter, total cells, eosinophil, macrophages, and other cells (neutrophils and lymphocytes) were counted. The levels of IL-4, IL-5, IL-6, IL-13, eotaxin (R&D system), and MUC5AC (Cusabio Biotech Co.) in BALF supernatant were measured using ELISA kits, according to the manufacturer's instructions. The absorbance was measured at 450 nm using a microplate reader (iMark^TM^, Bio-Rad Laboratories, Richmond, CA, United States).

### Analysis of Total IgE and OVA-specific IgE in Serum

The production of total IgE and OVA-specific IgE in serum were measured using ELISA kits (BioLegend) according to the manufacturer's protocols. The 96 microtiter plates were coated with OVA (10 μg/ml) and anti-IgE antibodies (anti-mouse IgE; 10 g/ml; BioLegend) in tris-buffered saline containing 0.05% Tween 20 (TBST), respectively, and incubated with a serum sample. The plates were washed four times, and 200 μL of o-phenylenediamine dihydrochloride (Sigma-Aldrich) was added to each well. The plates were incubated for 10 min in the dark, and the absorbance was measured at 450 nm using a microplate reader (Bio-Rad Laboratories).

### Histopathological Examination of Lungs

After BALF collection, the lung tissue was fixed in neutral-buffered formalin, embedded in paraffin, and cut into 4 μm thick sections. To estimate the amount of airway inflammation or mucus production, the lung tissue stained with hematoxylin and eosin (H&E) (BBC Biochemical, Mount Vemon, WA) or PAS (IMEB Inc.), respectively. Quantitative analysis of airway inflammation and mucus production was performed in a blinded manner with a light microscope (Leica Microsystem) at the 10× and 20× objective lens. The degree inflammation and mucus production of each slide were graded (0, no lesions; 1, minimal; 2, mild; 3, moderate; and, 4, severe) and the index levels of inflammation and mucus production were represented as mean ± SD.

### Immunoblotting in Lung Tissues

Lung tissues were homogenized (1/10, w/v) in a T-PER Tissue Protein Extraction Reagent (Thermo Scientific, Waltham, MA, United States) containing protease and phosphatase inhibitor cocktail (Thermo Scientific). The protein concentration for each samples was determined using a Bradford reagent (Bio-Rad Laboratories). Equal amounts of total protein (30 μg) were resolved by 4–12% SDS-polyacrylamide gel electrophoresis and transferred to polyvinyl difluoride membranes. Membranes were incubated with blocking solution (Thermo Scientific) followed by overnight incubation at 4°C with the following primary antibodies and dilutions: Erk, phospho (p)-Erk, JNK, p-JNK, p38MAPK, p-p38MAPK, IκB-α, p-IκB-α and β-actin (1:1,000 dilution; Cell Signaling Technology, Danvers, MA, United States), p65NF-κB, p-p65NF-κB and MUC5AC (1:1,000 dilution; Abcam, Cambridge, United Kingdom). The membranes were washed three times with Tris-buffered saline containing 0.05% Tween 20 (TBST) followed by incubation with a 1:10,000 dilution of horseradish peroxidase-conjugated secondary antibody (Cell Signaling Technology) for 1 h at room temperature. The blots were washed again three times with TBST. Protein bands were developed using an enhanced chemiluminescence kit (Thermo Scientific). Densitometric analysis for each protein band was determined using chemiluminescent scanner (LI-COR, Biosciences, Lincoln, NE, United States).

### Cell Culture and Cell Viability Assay

The NCI-H292 cells were maintained in RPMI 1640 media (Gibco, San Diego, CA, United States) supplemented with 10% heat-inactivated fetal bovine serum (FBS; Gibco) and antibiotics at 37°C in a 5% CO_2_ incubator. Cell viability after AH treatment was measured using a water-soluble tetrazolium salt 1 (WST-1; EZ-CyTox, Dogen, Republic of Korea) assay. The NCI-H292 cells were seeded in 96-well plates at a density of 5 × 10^4^ cells/well, incubated in RPMI1640 (0.1% FBS) and treated with different concentrations of AH (0, 20, 40, and 80 μg/ml). After 24 h of incubation, the effect of AH on cell viability was measured by the WST-1 assay, according to the manufacturer’s instructions. After the WST-1 solution was added to each well (10% of total volume) and incubated for 1 h, absorbance was measured using a microplate reader (Bio-Rad Laboratories) at 450 nm. The optical density of control cells was taken as 100% viability.

### Measurement of Pro-inflammatory Cytokine Levels in TNF-α-Stimulated NCI-H292 Cells

The cells (5×10^4^ cells/well) were incubated with AH at 0, 20, 40, and 80 μg/ml for 1 h before human recombinant TNF-α 30 ng/ml. The level of TNF-α and IL-6 in culture medium were determined at 24 h incubation, after TNF-α treatment and quantified using a competitive ELISA kit (R&D system) according to the manufacturer's instructions. The absorbance at 450 nm was measured in a microplate reader (Bio-Rad Laboratories).

### Immunoblotting in TNF-α-Stimulated NCI-H292 Cells

The cells (5×10^4^ cells/well) were incubated with AH (0, 20, 40, and 80 μg/ml) for 1 h, followed by incubation in the presence of TNF-α (30 ng/ml). After 1 h incubation, the cells were washed with PBS and protein was collected by using M-PER Extraction Reagents (Thermo Scientific) containing protease and phosphatase inhibitor cocktail. The expression levels of p-IκB-α, p-p65NF-κB and β-actin (loading control) were determined using immunoblotting as described above.

### Quantitative Real-Time Polymerase Chain Reaction (PCR)

The cells (5 × 10^4^ cells/well) were incubated with AH (0, 20, 40, and 80 μg/ml) followed by TNF-α (30 ng/ml) for 18 h. Thereafter, the cells were washed twice with PBS and total RNA was isolated using an RNA extraction kit (RNeasy^®^; Qiagen, Valencia, CA, United States). For real-time PCR, single-strand cDNA was synthesized from 1 μg of total RNA. Real-time PCR was conducted in triplicate with the CFX96 Touch^TM^ Real-time PCR detection system (Bio-Rad Laboratories), following the manufacturer’s specifications. A SensiFast^TM^ SYBR No-ROX kit (BioLine, Tauton, MA, United States) was used to prepare the substrate for PCR. The following primer sequences were used:

- IL-4 forward; 5′- ATG GGT CTC ACC TCC CAA CT -3′, IL-4 reverse; 5′- TAT CGC ACT TGT GTC CGT GG-3’ (Gene-Bank accession number: NM_172348.3).
- IL-5 forward; 5′- CAG GGA ATA GGC ACA CTG GA -3′, IL-5 reverse; 5′- TCT CCG TCT TTC TCC ACA C -3’ (Gene-Bank accession number: NM_000879.3).
- IL-13 forward; 5′- TGG TAT GGA GCA TCA ACC TGA C -3′, IL-13 reverse; 5′- GCA TCC TCT GGG TCT CG -3’ (Gene-Bank accession number: NM_001354993.2).
- GAPDH forward; 5′-ATC ACC ATC TTC CAG GAG CGA-3′, GAPDH reverse; 5′-AGG GGC CAT CCA CAG TCT T-3’ (Gene-Bank accession number: NM_001357943.2).

### Statistical Analysis

Data are expressed as the means ± standard deviation (SD). The statistical significance of the results was determined using analysis of variance, followed by a multiple comparison test with Tukey’s multiple comparison test. *p* values ≤0.05 were considered to be significant. Statistical analyses were performed using the GraphPad Prism 5 (GraphPad Software, CA, United States).

## Results

### Tentative Characterization of AH Extract

**FIGURE 1:**
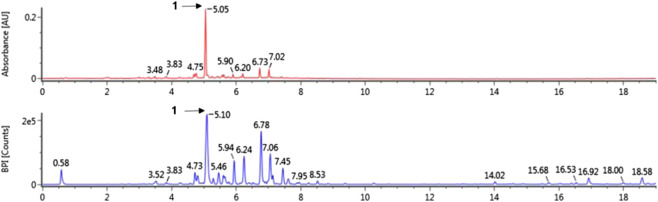
The UV (280 nm) and base peak intensity (BPI) chromatogram of *Alnus hirsuta* (Spach) Rupr. branch in negative mode using ultra-performance liquid chromatography-quadrupole-time of flight mass spectrometry (UPLC-QToF-MS).

The major peaks that were detected in *A. hirsuta* (Spach) Rupr. extract (Figure 1 and Table 1) were tentatively identified by comparison with previously literatures, as well as accurate mass and fragmentation pattern of mass spectra acquired in negative mode. The hirsutenone-pentoside (oregonin) has a molecular weight of 478 and its predominant peak at *m/z* 477 [M–H]^–^ generated distinctive ion at *m/z* 327 due to the loss of a pentose sugar residue (150 Da) (Riethmuller et al., 2015; Kang et al., 2020). Previous studies, oregonin was one of major diarylheptanoids in *A. hirsuta* (Lim et al., 2004; Ko et al., 2015). Therefore, the peak one tentatively identified as hirsutenone pentoside (oregonin).

**TABLE 1:** Tentatively identification of major peak detected in *Alnus hirsuta* (Spach) Rupr.

NO.	t_*R*_ (min)	Formula	Detected *m/z*	Exacted m/z	Error (ppm)	Fragments	Identification
1	5.05	C_24_H_30_O_10_	447.1779	447.1766	2.74	447, 327, 205	Hirsutenone-pentoside (oregonin)

### Effects of AH on Airway Resistance and Airway Hyperresponsiveness in OVA-Challenged Asthma Model

**FIGURE 2:**
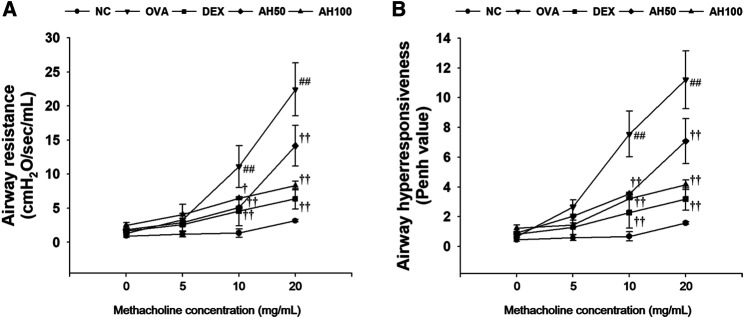
AH attenuates **(A)** airway resistance and **(B)** airway hyperresponsiveness in mice 24 h after final OVA challenge. NC: normal control mice; OVA: OVA-challenged mice; DEX: dexamethasone (3 mg/kg) + OVA-challenged mice; AH: AH (50 or 100 mg/kg) + OVA-challenged mice. The values are expressed as the means ± SD (n = 7/group). ^##^ *p* < 0.01, significantly different from NC group; ^†, ††^ *p* < 0.05, *p* < 0.01, significantly different from OVA-challenged group.

The OVA-challenged mice showed a significant elevation of airway resistance and airway hyperresponsiveness level in compared to normal controls at any concentration between 10 and 20 mg/ml of methacholine (Figure 2). However, AH-treated mice had showed lower airway resistance (Figure 2A) and airway hyperresponsiveness (Figure 2B) than the OVA-challenged mice. These reductions were similar to those observed in DEX-treated mice.

### Effects of AH on Inflammatory Cell Counts, Inflammatory Cytokines, Eotaxin and MUC5AC in the BALF of OVA-Challenged Asthma Model

**FIGURE 3:**
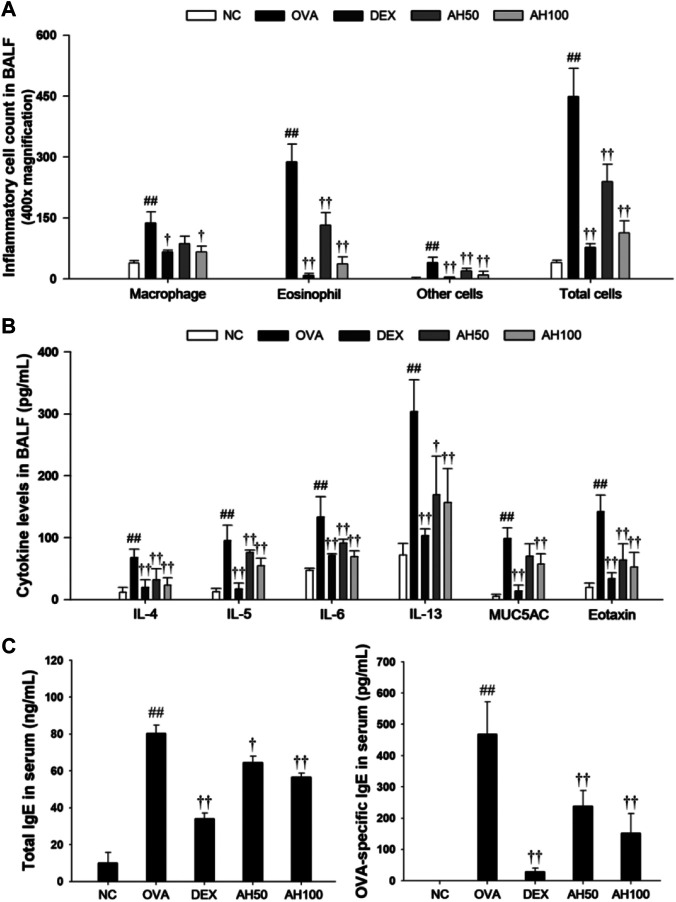
AH reduces inflammatory cell number, inflammatory cytokines, eotaxin, MUC5AC, total IgE and OVA-specific IgE in bronchoalveolar lavage fluid or serum of mice 48 h after final OVA challenge. **(A)** The inflammatory cells were attached on slide and stained with Diff-Quik stain reagent. **(B)** The levels of IL-4, IL-5, IL-6, IL-13, eotaxin and MUC5AC in the BALF were determined by ELISA. **(C)** Total IgE and OVA-specific IgE were determined using ELISA. NC: normal control mice; OVA: OVA-challenged mice; DEX: dexamethasone (3 mg/kg) + OVA-challenged mice; AH: AH (50 or 100 mg/kg) + OVA-challenged mice. The values are expressed as the means ± SD (n = 7/group). ^##^ *p* < 0.01, significantly different from NC group; ^††^ *p* < 0.01, significantly different from OVA-challenged group.

As show in Figure 3A, OVA-challenged mice showed significant increases in the number of eosinophils, macrophages, and other inflammatory cells compared to the normal controls (Figure 3A). However, DEX and AH treatments exhibited a significant decrease in inflammatory cells, in particular eosinophils and macrophages, in a dose-dependent manner compared with the OVA-challenged mice. The levels of Th2 cytokines and eotaxins in BALF were significantly higher in OVA-challenged mice compared to the normal controls (Figure 3B). In contrast, administration of DEX and AH decreased levels of Th2 cytokines and eotaxin compared to OVA-challenged mice. The MUC5AC level was significantly increased in OVA-challenged mice compared to the normal controls, whereas administration of DEX and AH showed a significant decrease in MUC5AC in the BALF compared with OVA-challenged mice in a dose-dependent manner.

### Effects of AH on Total IgE and OVA-specific IgE in the Serum of OVA-Challenged Asthma Model

The total IgE and OVA-specific IgE levels in the serum are shown in Figure 3C. Compared to the normal controls, OVA-challenged mice displayed a significant increase in total IgE and OVA-specific IgE in serum. However, DEX and AH treatments showed the marked reduction in the total IgE and OVA-specific IgE levels in the serum compared with those of the OVA-challenged mice in a dose-dependent manner.

### Effects of AH on Inflammatory Response and Mucus Production in the Lung Tissue of the OVA-Challenged Asthma Model

**FIGURE 4:**
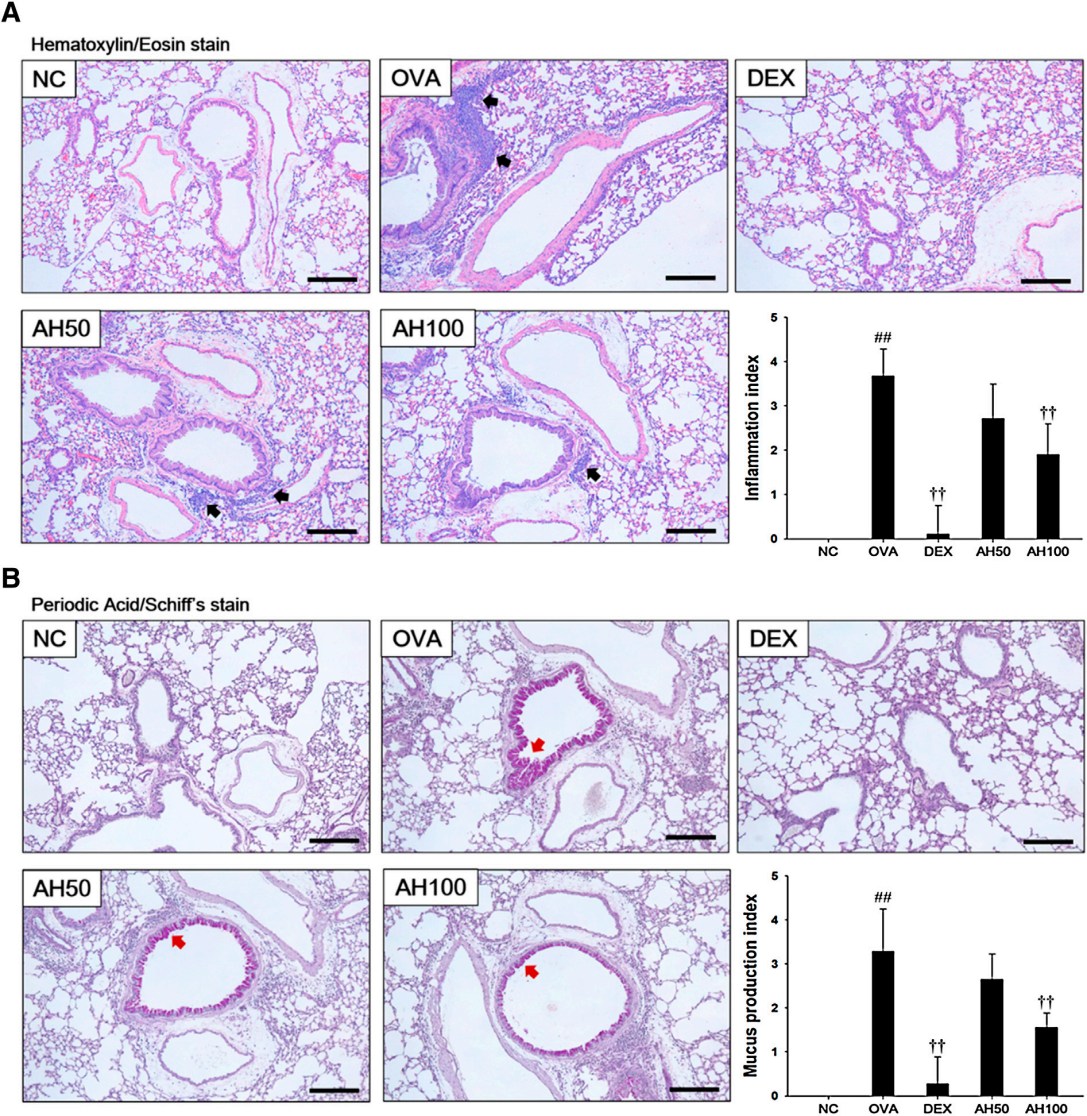
AH treatment attenuated airway inflammation and mucus production in lung of mice 48 h after final OVA challenge. Histopathological analysis on airway inflammation and mucus production was performed in the lung tissues by **(A)** H&E and **(B)** PAS staining. Black-colored arrow indicated the lesion of inflammatory cells infiltration. Red-colored arrow indicated mucus production. Scale bars, 50 μm. NC: normal control mice; OVA: OVA-challenged asthma mice; DEX: dexamethasone (3 mg/kg) + OVA-challenged asthma mice; AH: AH (50 or 100 mg/kg) + OVA-challenged asthma mice. The values expressed as the means ± SD (n = 7/group). ^##^ *p* < 0.01, significantly different from NC group; ^††^ *p* < 0.01, significantly different from OVA-challenged group.

The OVA-challenged mice led to marked increases in infiltration of inflammatory cells into the peribronchiolar and perivascular lesions in the lung tissue (Figure 4A). The DEX group significantly attenuated infiltration of inflammatory cells. Moreover, AH groups showed a dose-dependent decrease in inflammatory cell infiltration compared with the OVA-challenged mice. Lung sections from OVA-challenged mice stained with PAS exhibited a goblet cell hyperplasia and overproduction of mucus relative to normal controls (Figure 4B). In contrast, sections from DEX and AH groups exhibited a reduction in hyperplasia and mucus overproduction in the bronchial airway compared with OVA-challenged mice.

### Effects of AH on MAPKs/p65NF-κB and MUC5AC in the Lung Tissue of the OVA-Challenged Asthma Model

**FIGURE 5:**
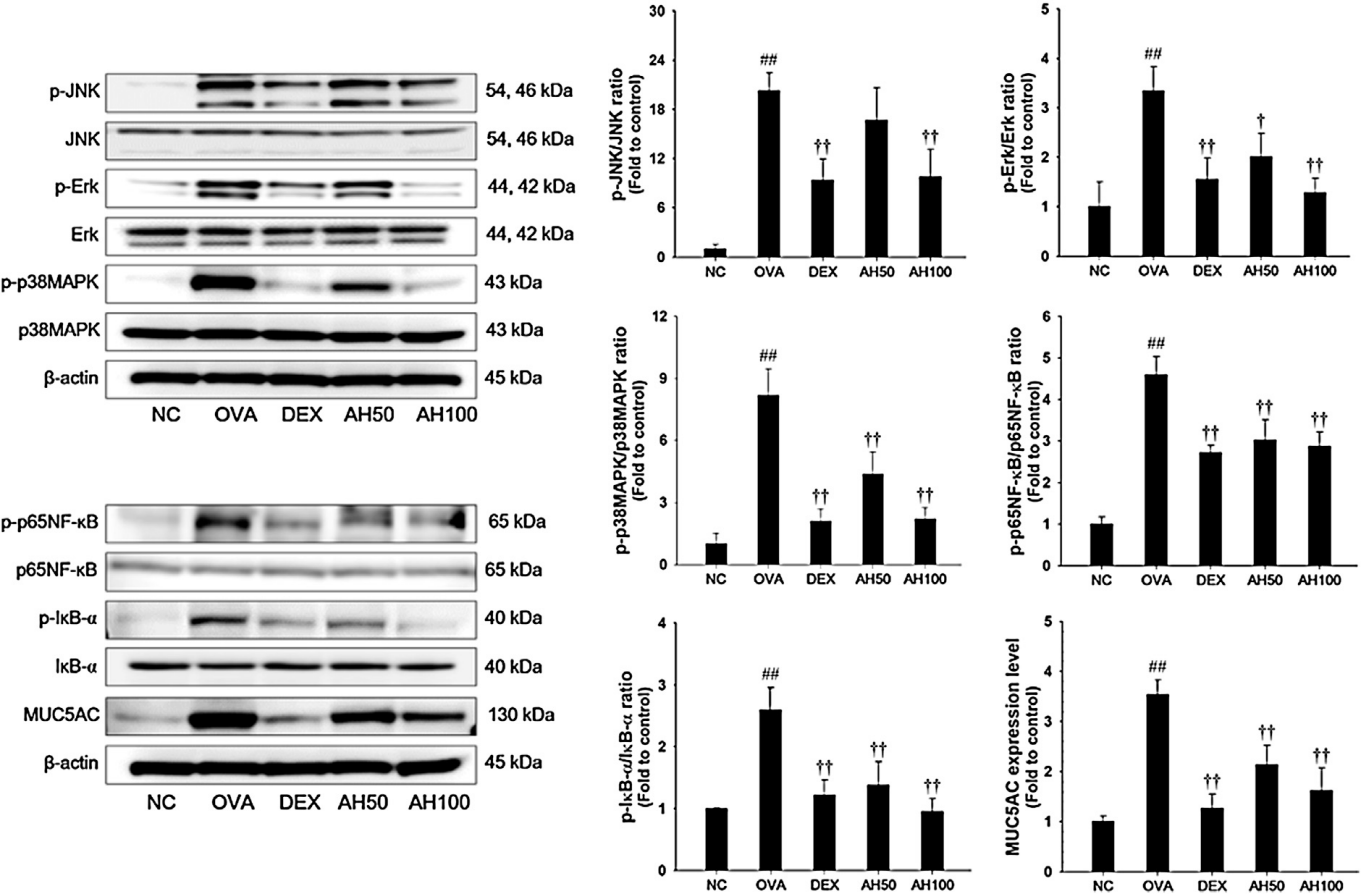
AH inhibited phosphorylation of MAPKs/p65NF-κB and expression of MUC5AC in lung of mice 48 h after final OVA challenge. The protein levels of MAPKs/p65NF-κB and MUC5AC in the total lung tissues were determined by western blot analysis. β-actin was used to confirm equal protein loading. NC: normal control mice; OVA: OVA-challenged asthma mice; DEX: dexamethasone (3 mg/kg) + OVA-challenged asthma mice; AH: AH (50 or 100 mg/kg) + OVA-challenged asthma mice. The values are expressed as the means ± SD (n = 7/group). ^##^ *p* < 0.01, significantly different from NC group; ^†,††^ *p* < 0.05, *p* < 0.01, significantly different from OVA-challenged group.

The underlying mechanism of AH, we determined the effects of AH on the MAPKs, p65 NF-κB and MUC5AC expression. As shown in Figure 5, the OVA-challenged mice had a remarkable increase in the phosphorylation of MAPKs/p65NF-κB, with elevation of MUC5AC expression in the lung tissue compared to levels in normal control mice. However, treatment of DEX and AH significantly inhibited the phosphorylation of MAPKs/p65NF-κB with reduction of MUC5AC expression in the lung tissues compared with the OVA-challenged mice.

### Effects of AH on Pro-inflammatory Cytokine, Th2 Cytokines Production and p65NF-κB in TNF-α-Stimulated NCI-H292 Cells

**FIGURE 6:**
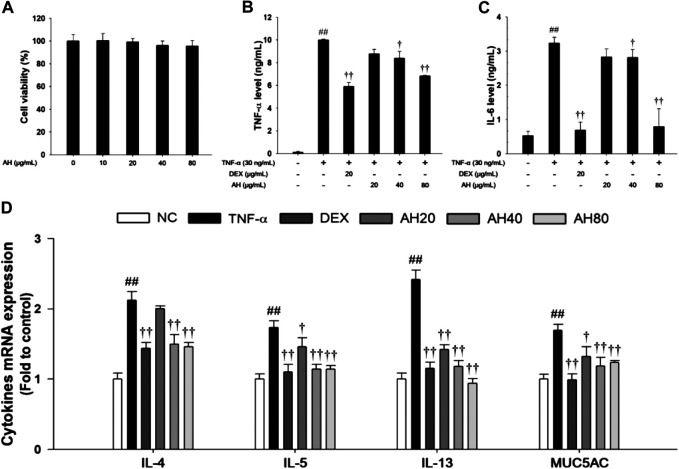
Effects of AH on cell viability, inflammatory cytokines (TNF-α, IL-6), Th2 cytokines (IL-4, IL-5 and IL-13) and MUC5AC in TNF-α-stimulated NCI-H292 cells. The **(A)** cell viability was measured using a WST-1 reagent, and AH was treated with 10, 20, 40 and 80 μg/ml for 24 h. The levels of **(B)** TNF-α and **(C)** IL-6 was determined by ELISA. The culture medium were changed RPMI1640 (0.1% FBS) and treated with AH (20, 40 and 80 μg/ml) for 1 h and incubated with TNF-α (30 ng/ml) for 24 min (TNF-α, IL-6). **(D)** The mRNA levels of IL-4, IL-5, IL-13 and MUC5AC were measured by real-time PCR in TNF-α-stimulated NCI-H292 cells. Cells were treated with AH (20, 40 and 80 μg/ml) for 1 h and incubated with TNF-α (30 ng/ml) for 18 h. NC: Normal control NCI-H292 cells; TNF-α: TNF-α-stimulated NCI-H292 cells; DEX: dexamethasone (20 μg/ml) + TNF-α-stimulated NCI-H292 cells; AH: AH (20, 40 and 80 mg/kg) + TNF-α-stimulated NCI-H292 cells. The values are expressed as the means ± SD (n = 3). ^##^ *p* < 0.01, Significantly different from control; ^†,††^ *p* < 0.05, *p* < 0.01, significantly different from TNF-α-treated group.

**FIGURE 7:**
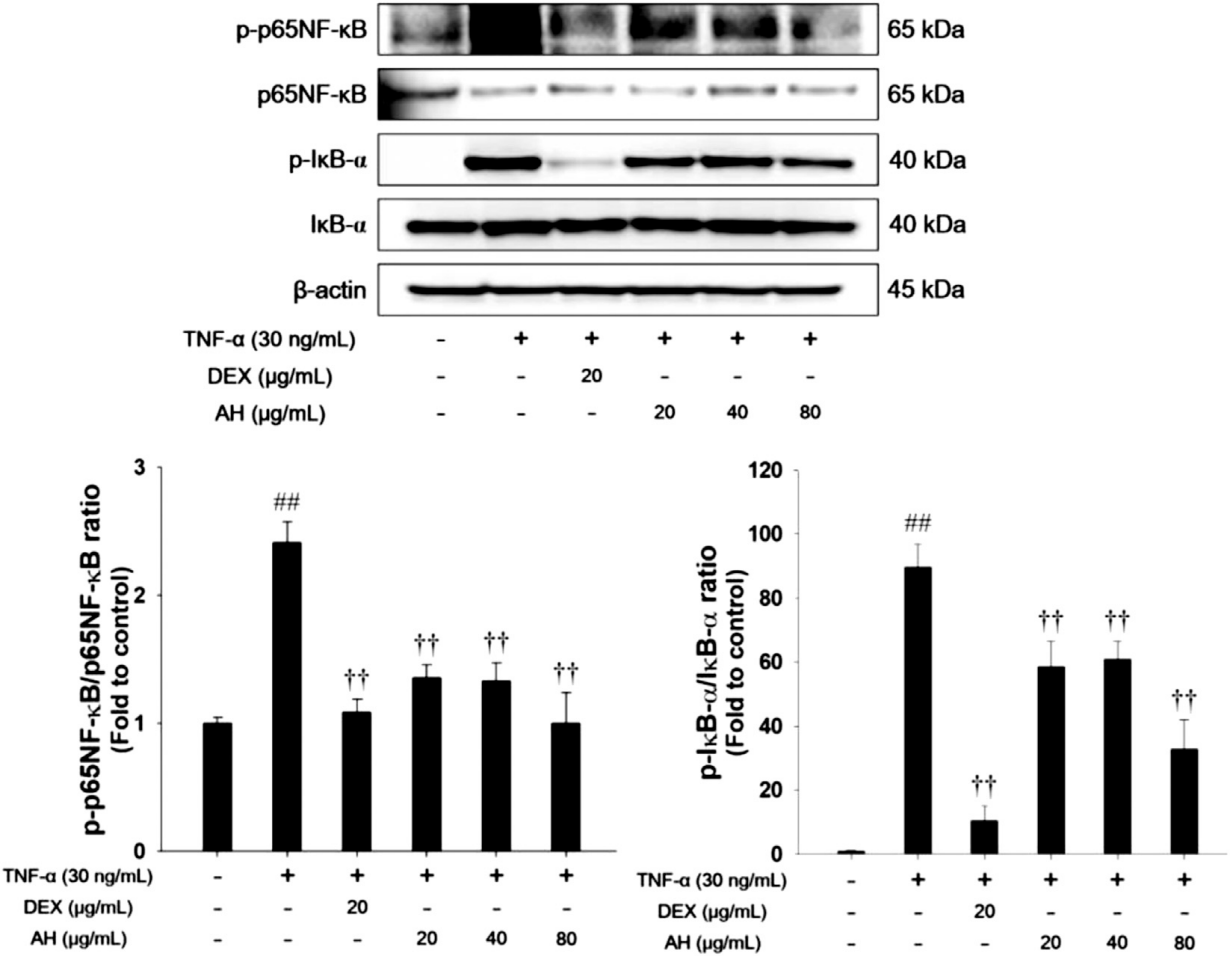
AH inhibited phosphorylation of p65NF-κB and IκB-α in TNF-α-stimulated NCI-H292 cells. β-actin was used to confirm equal protein loading. The culture medium were changed RPMI1640 (0.1% FBS) and treated with AH (20, 40 and 80 μg/ml) for 1 h and incubated with TNF-α (30 ng/ml) for 30 min. The values are expressed as the means ± SD (n = 3). ^##^ *p* < 0.01, Significantly different from control; ^††^ *p* < 0.01, significantly different from TNF-α-treated group.

Based on the results of cytotoxicity analysis, nontoxic concentrations (20, 40, and 80 μg/ml) of AH were employed in the present study (Figure 6A). TNF-α (30 ng/ml) treatment markedly increased the TNF-α level compared to levels in non-stimulated cells. However, AH-treated cells had a significant decrease in levels of TNF-α and IL-6 in a concentration-dependent manner relative to TNF-α-stimulated cells (Figures 7B,C). By analyzing the mRNA levels of the Th2 cytokines, IL-4, IL-5 and IL-13, and MUC5AC, we found that the TNF-α-stimulated cells exhibited a significant increase in the mRNA levels of IL-4, IL-5, IL-13 and MUC5AC in NCI-H292 cells. Compared to TNF-α-stimulated cells, cells treated with AH had a significant reduction in the mRNA levels of the Th2 cytokines and MUC5AC in a concentration-dependent manner (Figure 6D).

As shown in Figure 7, TNF-α treatment increased phosphorylation of p65NF-κB and IκB-α in TNF-α-stimulated NCI-H292 cells. However, treatment with AH significantly decreased phosphorylation of p65NF-κB and IκB-α.

## Discussion

Allergic asthma is an important pulmonary disease that affects 275 million people worldwide (Huang et al., 2017). The characteristics of asthma include chronic airway inflammation, mucus overproduction and airway hyperresponsiveness, leading to airway obstruction (Halwani et al., 2010; Liu et al., 2014). Recently, there has been increased research on herbal medicines that have anti-inflammatory properties for treatment of allergic asthma (Jung et al., 2020; Liou et al., 2020). In this study, we investigated the effects of an AH extract on airway inflammation using TNF-α-stimulated NCI-H292 cells and an OVA-challenged asthmatic mouse model. Treatment with AH not only inhibited the production of TNF-α and IL-6, but also suppressed expression of IL-4, IL-5, IL-13, and MUC5AC in TNF-α-stimulated NCI-H292 cells. AH treatment markedly decreased the infiltration of inflammatory cells, particularly eosinophils and macrophages, and suppressed Th2 cytokine, eotaxin, and MUC5AC level in the BALF, as well as OVA-specific IgE in the serum of OVA-challenged asthmatic mice. AH treatment also attenuated airway resistance/hyperresponsiveness and mucus production in OVA-challenged mice. Moreover, AH effectively downregulated the phosphorylation of MAPKs/p65NF-κB, and the expression of MUC5AC in lung tissue of OVA-challenged asthmatic mice.

Eosinophilia is a hallmark of allergic asthma. It is associated with the activation of inflammatory cells such as Th2 cells and eosinophils, which produce cytokines, including IL-4, IL-5 and IL-13, and chemokine (eotaxin). Th2 cytokines induce IgE switching, eosinophil maturation, and goblet cell hyperplasia, resulting in mucus overproduction and airway hyperresponsiveness in allergic asthma (Leon, 2017). Many studies have demonstrated that Th2 cytokines play a key role in the development and maintenance of allergic asthma (Ko et al., 2017; Gandhi et al., 2019). Thus, modulation of Th2 cytokines may constitute a therapeutic target in allergic asthma. In this study, the AH treatment significantly reduced Th2 cytokines and eotaxins in BALF and decreased level of OVA-specific IgE in serum from OVA-challenged mice. Moreover, airway resistance/hyperresponsiveness, airway inflammation, and mucus overproduction in OVA-challenged mice were suppressed by AH treatment. It has been demonstrated that herbal medicines with anti-inflammatory effects markedly attenuate airway inflammation and airway hyperresponsiveness via inhibition of Th2 cytokine production in the OVA-challenged asthma model (Asma et al., 2017; Sung et al., 2019). Therefore, these findings suggest that AH treatment effectively inhibited OVA-challenged allergic asthma via suppression of Th2 cytokines.

The inflammatory response during allergic asthma is due to activation of the major transcription factor NF-κB (Wang et al., 2017). Upon activation, IκB kinase phosphorylates NF-κB. The p65-p50 dimer of NF-κB is then translocated into the nucleus where it binds to DNA to regulate the expression of Th2 cytokines and MUC5AC in allergic asthma (Xie et al., 2015). MUC5AC is a major component of airway mucus. In asthmatic patients, MUC5AC expression is elevated by goblet cells, leading to airway limitation (Shin et al., 2019b, Song et al., 2005). In addition, MAPKs also regulate the expression of Th2 cytokines, and can affect the differentiation of inflammatory cells, such as Th2 cells and eosinophils. In a previous study, activation of MAPKs/NF-κB signaling was considered an important factor in the development of allergic asthma (Chauchan et al., 2018). In our study, AH-treated asthmatic mice exhibited a significant decrease in the phosphorylation of MAPKs/p65NF-κB and MUC5AC expression. These results are consistent with the results of our *in vitro* experiments. Several studies reported that blocking of MAPKs/NF-κB signaling led to significant decreases in Th2 cytokines and a reduction in mucus overproduction, leading to reduction of airway inflammation in the OVA-challenged asthmatic mouse model (Asma et al., 2017; Chauchan et al., 2018; Fengjuan et al., 2019). Thus, our results indicate that anti-asthmatic effects of AH treatment on OVA-challenged airway inflammation and mucus overproduction may be associated with inhibition of MAPKs/NF-κB, resulting in suppression of MUC5AC expression.

AH is widespread in the Eastern Asia and is used in traditional medicine as a remedy for hemorrhage, fever, and diarrhea (Lee, 1996). The AH branches were collected from Gangwon Province (Gangwon-do, South Korea) in April of 2016. The AH extracted with 99.9% methyl alcohol at room temperature and collected from The Korea Plant Extract Bank of KRIBB (PB2357.6). The AH is reported to contain rubranoside A, hirsutanonol 5-O-β-D-glucopyranoside, platyphyllenone and oregonin (Park et al., 2010). According to previous studies in LPS-stimulated RAW264.7 cells, the antioxidant effect of AH may be due to the suppression of ROS and NO production, while its anti-inflammatory properties of AH include inhibition of inflammatory mediators and cytokines via downregulation of NF-κB (Hu and Wang, 2011; Jin et al., 2007). In our study, oregonin was the major compound of methanolic extract of AH. Oregonin inhibits the production of Th2 cytokines and IgE, as well as eosinophils in atopic dermatitis in NC/Nga mice (Choi et al., 2010). In addition, oregonin downregulates iNOS and COX-2, and decreases NO production by suppressing the NF-κB pathway (Lee et al., 2005). However, there are no reports on the protective effects of AH against allergic asthma in an *in vivo* model. Based on our findings, treatment with a methanolic extract of AH effectively attenuates airway inflammation and mucus overproduction by inhibiting the phosphorylation of MAPKs/p65NF-κB.

In this study, AH showed protective effects in OVA-challenged asthmatic mouse model. However, these results have some limitations in our study. First, the AH was administered before OVA challenge, as opposed to after onset of asthmatic responses. This strategy would mimic a preventive procedure rather than curative procedure. Second, it has not yet been determined that the major compounds in AH have anti-inflammatory activities in the allergic asthma model. Thus, further studies will be investigated to curative effects via chronic allergic asthma model (Nials and Uddin, 2008) and evaluate to anti-inflammatory activities of oregonin in allergic asthma model for therapeutic agent of AH.

## Conclusion

In conclusion, to the best of our knowledge, this is the first study to demonstrate that treatment with an AH attenuates airway inflammation and mucus hypersecretion by reducing Th2 cytokine release and blocking of MAPKs/p65NF-κB phosphorylation in lung tissue from OVA-challenged asthmatic mouse model. These findings suggest that methanolic extract of AH can markedly protect against allergic asthma. Thus, our results suggest that AH can be developed as a useful medicine for the treatment of allergic asthma.

## Data Availability

The original contributions presented in the study are included in the article/Supplementary Material, further inquiries can be directed to the corresponding authors.

## References

[B1] Alam R. Gorska M. M. (2011). Mitogen-activated protein kinase signaling and ERK1/2 bistability in asthma. Clin. Exp. Allergy 41, 149–159. 10.1111/j.1365-2222.2010.03658.x 21121982 PMC3115726

[B2] Asma I. Muhammad S. Arham S. Hira S. Khadija S. Aqeel J. (2017). *Carica papaya* ameliorates allergic asthma via downregulation of IL-4, IL-5, eotaxin, TNF-α, NF-κB, and iNOS levels. Pytomedicine 15 (32), 1–7. 10.1016/j.phymed.2017.04.009 28732802

[B3] Bang M. A. Seo J. H. Seo J. W. Jo G. H. Jung S. K. Yu R. (2015). Bacillus subtilis KCTC 11782BP-produced alginate oligosaccharide effectively suppresses asthma via T-helper cell type 2-related cytokines. PLoS One 10, e0117524 10.1371/journal.pone.0117524 25658604 PMC4319839

[B5] Cai B. Seong K. J. Bae S. W. Chun C. Kim W. J. Jung J. Y. (2018). A synthetic diosgenin primary amine derivative attenuates LPS-stimulated inflammation via inhibition of NF-κB and JNK MAPK signaling in microglial BV2 cells. Int. Immunopharm. 61, 204–214. 10.1016/j.intimp.2018.05.021 29890414

[B6] Calderon M. A. Casale T. B. Nelson H. S. Demoly P. (2013). An evidence-based analysis of house dust mite allergen immunotherapy: a call for more rigorous clinical studies. J. Allergy Clin. Immunol. 132, 1322–1336. 10.1016/j.jaci.2013.09.004 24139829

[B7] Chauchan P. S. Singh D. K. Dash D. Singh R. (2018). Intranasal curcumin regulates chronic asthma in mice by modulating NF-κB and MAPK signaling. Pytomedicine 51, 29–38. 10.1016/j.phymed.2018.06.022 30466625

[B8] Choi S. E. Jeong M. S. Kang M. J. Lee D. K. Joo S. S. Lee C. S. (2010). Effect of topical application and intraperitoneal injection of oregonin on atopic dermatitis in NC/Nga mice. Exp. Dermatol. 19 (8), e37–e43 10.1111/j.1600-0625.2009.00961.x 19849716

[B9] Ciriaco M. Ventrice P. Russo G. Scicchitano M. Mazzitello G. Scicchitano F. (2013). Corticosteroid-related central nervous system side effects. J. Pharmacol. Pharmacother. 4, S94–S98. 10.4103/0976-500X.120975 24347992 PMC3853679

[B10] Costa R. S. Carneiro T. C. B. Cerqueira-Lima A. T. Queiroz N. V. Alcantara-Neves N. M. Ponres-de-Carvalhom L. C. (2012). *Ocimum gratissimum* Linn. and rosmarinic acid, attenuate eosinophilic airway inflammation in an experimental model of respiratory allergy to Blomia tropicalis. Int. Immunopharm. 13, 126–134. 10.1016/j.intimp.2012.03.012 22465960

[B11] Fengjuan Y. Rui L. Mengying H. Xiaojuan R. Liping B. Lei X. (2019). JAX2, an ethanol extract of *Hyssopus cuspidatus* Boriss, can prevent bronchial asthma by inhibiting MAPK/NF-κB inflammatory signaling. Pytomedicine 57, 305–314. 10.1016/j.phymed.2018.12.043 30807985

[B12] Gandhi G. R. Neta M. T. S. L. Sathiyabama R. G. Quintans J. D. S. S. Silva A. M. D. O. E. Araujo A. A. S. (2019). Flavonoids as Th1/Th2 cytokines immunomodulators: a systemic review of studies on animal models. Pytomedicine 15 (44), 74–84. 10.1016/j.phymed.2018.03.057 29895495

[B13] Halwani R. Al-Muhsen S. Hamid Q. (2010). Airway remodeling in asthma. Curr. Opin. Pharmacol. 10, 236e245 10.1016/j.coph.2010.06.004 20591736

[B14] Hu W. Wang M. H. (2011). Antioxidative activity and anti-inflammatory effects of diarylheptanoids isolated from *Alnus hirsuta* . JWRS 57 (4), 323–330. 10.1007/s10086-010-1170-x

[B15] Huang C. C. Chang P. H. Wu P. W. Wang C. H. Fu C. H. Huang C. C. (2017). Impact of nasal symptoms on the evaluation of asthma control. Medicine 96, e6147 10.1097/MD.0000000000006147 28225496 PMC5569424

[B16] Jin W. Y. Cai X. F. Na M. Lee J. J. Bae K. (2007). Diarylheptanoids from *Alnus hirsuta* inhibit the NF-kB activation and NO and TNF-a production. Biol. Pharm. Bull. 30 (4), 810–813. 10.1248/bpb.30.810 17409527

[B17] Jung T. Y. Lee A. Y. Song J. H. Lee M. Y. Lim J. O. Lee S. J. (2020). *Scrophularia koraiensis* Nakai attenuates allergic airway inflammation via suppression of NF-κB and enhancement of Nrf2/HO-1 signaling. Antioxidants 9 (2), 99 10.3390/antiox9020099 PMC7070852 31991647

[B18] Kang K. B. Woo S. Ernst M. Hooft J. J. J. Nothias L. Silva R. R. (2020). Assessing specialized metabolite diversity of *Alnus* species by a digitized LC-MS/MS data analysis workflow. Phytochemistry 173, 112292 10.1016/j.phytochem.2020.112292 32062198

[B19] Kim M. Kim S. Min J. Kwon O. Park M. Park J. (2019). Anti-inflammatory effects of linalool on ovalbumin-induced pulmonary inflammation. Int. Immunopharm. 74, 105706 10.1016/j.intimp.2019.105706 31254955

[B20] Ko E. K. Choi H. Jin H. Choi S. (2015). Oregonin from the stems and leaves of Korean *Alnus* species (Betulaceae). J. Chem. Pharmaceut. Res. 7 (4), 234–238

[B21] Ko J. Shin N. Park S. Cho Y. Kim J. Seo C. (2017). Genipin inhibits allergic responses in ovalbumin-induced asthmatic mice. Int. Immunopharm. 53, 49–55. 10.1016/j.intimp.2017.10.010 29035815

[B22] LaVan D. A. Lynn D. M. Langer R. (2002). Moving smaller in drug discovery and delivery. Nat. Rev. Drug Discov. 1, 77–84. 10.1038/nrd707 12119612

[B23] Lee C. J. Lee S. S. Chen S. C. Ho F. M. Lin W. W. (2005). Oregonin inhibits lipopolysaccharide-induced iNOS gene transcription and upregulates HO-1 expression in macrophages and microglia. Br. J. Pharmacol. 146, 378–388. 10.1038/sj.bjp.0706336 16025135 PMC1576284

[B24] Lee J. W. Kim Y. I. Im C. N. Kim S. W. Kim S. J. Min S. (2017). Grape seed proanthocyanidin inhibits mucin synthesis and viral replication of suppression of AP-1 and NF-κB via p38 MAPKs/JNK signaling pathways in respiratory syncytial virus-infected A549 cells. J. Agric. Food Chem. 65, 4472–4483. 10.1021/acs.jafc.7b00923 28502165

[B25] Lee S. J. (1996). Korea folk medicine. Seoul, Korea: Seoul National University Publishing Center Press, 40

[B26] Lee S. Y. Bae C. S. Seo N. Na C. Yoo H. Y. Oh D. (2019). *Camellia Japonica* oil suppressed asthma occurrence via GATA-3 & IL-4 pathway and its effective and major component is oleic acid. Phytomedicine 57, 84–94. 10.1016/j.phymed.2018.12.004 30668326

[B27] Leon B. (2017). T cells in allergic asthma: key players beyond the Th2 pathway. Curr. Allergy Asthma Rep. 17 (7), 43 10.1007/s11882-017-0714-1 28555329

[B28] Lim H. Kim M. Kim H. Shim J. Kim G. Choi H. (2004). Quantitative determination of diarylheptanoid compounds from Korean *Alnus* . Korean J. Pharmacogn. 35 (4), 384–387

[B29] Liou C. J. Chen Y. L. Yu M. C. Yeh K. W. Shen S. C. Huang W. C. (2020). Sesamol alleviates airway hyperresponsiveness and oxidative stress in asthmatic mice. Antioxidants 9 (4), 295 10.3390/antiox9040295 PMC7222203 32244835

[B30] Liu F. Zhao Y. Liu Y. Q. Liu Y. Sun J. Huang M. M. (2014). Asthma and asthma related symptoms in 23,326 Chinese children in relation to indoor and outdoor environmental factors: the seven northeastern cities (SNEC) Study. Sci. Total Environ., 497–498. 10.1016/j.scitotenv.2014.07.096 25112820

[B31] Nials A. T. Uddin S. (2008). Mouse models of allergic asthma: acute and chronic allergen challenge. Dis. Model Mech. 1 (4–5), 213–220. 10.1242/dmm.000323 19093027 PMC2590830

[B32] Park D. Kim H. J. Jung S. Y. Yook C. S. Jin C. Lee Y. S. (2010). A new diarylheptanoid glycoside from the stem bark of *Alnus hirsuta* and protective effects of diarylheptanoid derivatives in human HepG2 cells. Chem. Pharm. Bull. 58 (2), 238–241. 10.1248/cpb.58.238 20118587

[B33] Park J. W. Lee I. C. Shin N. R. Jeon C. M. Kwon O. K. Ko J. W. (2016). Copper oxide nanoparticles aggravate airway inflammation and mucus production in asthmatic mice via MAPK signaling. Nanotoxicology 10 (4), 445–452. 10.3109/17435390.2015.1078851 26472121

[B34] Peebles R. S. Aronica M. A. (2019). Proinflammatory pathways in the pathogenesis of asthma. Clin. Chest Med. 40 (1), 29–50. 10.1016/j.ccm.2018.10.014 30691715 PMC6364573

[B35] Perret J. L. Dharmage S. C. Matheson M. C. Johns D. P. Gurrin L. C. Burgess J. A. (2013). The interplay between the effects of lifetime asthma, smoking, and atopy on fixed airflow obstruction in middle age. Am. J. Respir. Crit. Care Med. 187, 42–48. 10.1164/rccm.201205-0788OC 23155143

[B36] Riethmuller E. Toth G. Alberti A. Vegh K. BurliniKonczol I. A. (2015). First characterisation of flavonoid- and diarylheptanoid-type antioxidant phenolics in *Corylus maxima* by HPLC-DAD-ESI-MS. J. Pharmaceut. Biomed. Anal. 25 (107), 159–167. 10.1016/j.jpba.2014.12.016. 25594894

[B37] Saxena A. Yadav D. Maurya A. K. Kumar A. Mohanty S. (2016). Diarylheptanoids from *Alnus nepalensis* attenuates LPS-induced inflammation in macrophages and endotoxic shock in mice. Int. Immunopharm. 30, 129–136. 10.1016/j.intimp.2015.12.002 26679675

[B38] Schatz M. Rosenwasser L. (2014). The allergic asthma phenotype. J. Allergy Clin. Immunol. Pract. 2, 645–648. 10.1016/j.jaip.2014.09.004 25439351

[B39] Shalaby K. H. Gold L. G. Schuessler T. F. Martin J. G. Robichaud A. (2010). Combined forced oscillation and forced expiration measurements in mice for the assessment of airway hyperresponsiveness. Respir. Res. 11, 82 10.1186/1465-9921-11-82 20565957 PMC2904286

[B40] Shin I. Shin N. Jeon C. Kwon O. Hong J. Kim H. (2019a). *Thuha orientalis* reduces airway inflammation in ovalbumin-induced allergic asthma. Mol. Med. Rep. 12, 4640–4646. 10.3892/mmr.2015.3910 26063078

[B41] Shin N. R. Kwon H. J. Ko J. W. Kim J. S. Lee I. C. Kim J. C. (2019b). S-allyl cysteine reduces eosinophilic airway inflammation and mucus overproduction on ovalbumin-induced allergic model. Int. Immunopharm. 68, 124–130. 10.1016/j.intimp.2019.01.001 30622029

[B42] Shin N. R. Shin I. S. Song H. H. Hong J. M. Kwon O. K. Jeon C. M. (2015). *Callicarpa japonica* Thunb. reduces inflammatory responses: a mouse model of lipopolysaccharide-induced acute lung injury. Int. Immunopharm. 26, 174–180. 10.1016/j.intimp.2015.01.025 25662753

[B43] Shin N. Ryu H. Ko J. Park S. Yuk H. Kim H. (2017). *Artemisia argyi* attenuates airway inflammation in ovalbumin-induced asthmatic animals. J. Ethnopharmacol. 209, 108–115. 10.1016/j.jep.2017.07.033 28735728

[B44] Song J. S. Cho K. S. Yoon H. K. Moon H. S. Park S. H. (2005). Neutrophil elastase causes MUC5AC mucin synthesis via EGF receptor, ERK and NF-kB pathways in A549 cells. Korea J. Intern. Med. 20, 275–283. 10.3904/kjim.2005.20.4.275 PMC3891072 16491824

[B45] Sung Y. Kim S. Yuk H. J. Yang W. Lee Y. M. Son E. (2019). *Siraitia grosvenorii* residual extract attenuates ovalbumin-induced lung inflammation by down-regulating IL-4, IL-5, IL-13, IL-17 and MUC5AC expression in mice. Phytomedicine 61, 152835 10.1016/j.phymed.2019.152835 31035047

[B46] Tian C. Zhang P. Yang J. Zhang Z. Wang H. Guo Y. (2019). The protective effect of the flavonoid fraction of *Abutilon theophrasti* Medic. leaves on LPS-induced acute lung injury in mice via the NF-κB and MAPK signaling pathways. Biomed. Pharmacother. 109, 1024–1031. 10.1016/j.biopha.2018.10.197 30551352

[B47] Wang X. Yang X. Li Y. Wang X. Zhang Y. Dai X. (2017). Lyn kinase represses mucus hypersecretion by regulating IL-13-induced endoplasmic reticulum stress in asthma. EBioMedicine 15, 137–149. 10.1016/j.ebiom.2016.12.010 28024734 PMC5233819

[B48] Wise J. (2014). Corticosteroids for asthma may suppress growth in children in first year of treatment, researchers say. BMJ 349, g4623 10.1136/bmj.g4623 25035241

[B49] Xie T. Luo G. Zhang Y. Wang X. Wu M. Li G. (2015). Rho-kinase inhibitor fasudil reduces allergic airway inflammation and mucus hypersecretion by regulating STAT6 and NFkappaB. Clin. Exp. Allergy 45 (12), 1812–1822. 10.1111/cea.12606 26245530

[B50] Zhang Q. Wang L. Chen B. Zhuo Q. Bao C. Lin L. (2017). Propofol inhibits NF-kappaB activation to ameliorate airway inflammation in ovalbumin (OVA)-induced allergic asthma mice. Int. Immunopharm. 51, 158–164. 10.1016/j.intimp.2017.08.015 28843179

